# Early onset of breast cancer in a group of British black women

**DOI:** 10.1038/sj.bjc.6604174

**Published:** 2008-01-08

**Authors:** R L Bowen, S W Duffy, D A Ryan, I R Hart, J L Jones

**Affiliations:** 1 Centre for Tumour Biology, Institute of Cancer and CR-UK Clinical Centre, Barts and The London, Queen Mary's School of Medicine and Dentistry, John Vane Science Centre, Charterhouse Square, London EC1M 6BQ, UK; 2 Cancer Research UK, Centre for Epidemiology, Mathematics and Statistics, Wolfson Institute of Preventive Medicine, Charterhouse Square, London EC1M 6BQ, UK; 3 Department of Histopathology, St Bartholomew's Hospital, West Smithfield, London EC1A 7BE, UK

**Keywords:** British black women, invasive breast cancer, age distributions, clinicopathological features, triple negative, socioeconomic status

## Abstract

Since there are no published data on breast cancer in British black women, we sought to determine whether, like African-American women, they present at a younger age with biologically distinct disease patterns. The method involved a retrospective review of breast cancer to compare age distributions and clinicopathological features between black women and white women in the UK, while controlling for socioeconomic status. All women presented with invasive breast cancer, between 1994 and 2005, to a single East London hospital. Black patients presented significantly younger (median age of 46 years), than white patients (median age of 67 years (*P*=0.001)). No significant differences between black and white population structures were identified. Black women had a higher frequency of grade 3 tumours, lymph node-positive disease, negative oestrogen receptor and progesterone receptor status and basal-like (triple negative status) tumours. There were no differences in stage at presentation; however, for tumours of ⩽2 cm, black patients had poorer survival than white patients (HR=2.90, 95% CI 0.98–8.60, *P*=0.05). Black women presented, on average, 21 years younger than white women. Tumours in younger women were considerably more aggressive in the black population, more likely to be basal-like, and among women with smaller tumours, black women were more than twice as likely to die of their disease. There were no disparities in socioeconomic status or treatment received. Our findings could have major implications for the biology of breast cancer and the detection and treatment of the disease in black women.

While the incidence of breast cancer in women of African descent is lower than that of their white counterparts, paradoxically, the age adjusted breast cancer rates are higher (Elledge *et al*, 1994; Brawley, 2002). Black women have been shown to present with a more advanced stage of disease and at a younger age in published American and African studies (Adebamowo and Adekunle, 1999; Joslyn and West, 2000). There are, however, no published data on breast cancer presentation in the British black population.

From the American studies it appears that up to 35% of black breast cancer patients are under 50 years old, compared with only 20% of white women with breast cancer (Elledge *et al*, 1994; Newman and Alfonso, 1997; El-Tamer *et al*, 1999). African-American (A-A) women had significantly larger tumours, lower rates of localised disease and higher rates of oestrogen receptor (ER) and progesterone receptor (PgR) negativity, all of which confer a poorer prognosis (Elledge *et al*, 1994; Joslyn and West, 2000; Newman *et al*, 2002; Jones *et al*, 2004; Gukas *et al*, 2005). These A-A women were more likely than white patients to present with poorly differentiated and medullary-like tumours, and they might have up to double the incidence of inflammatory carcinoma, the most aggressive form of breast cancer (Chang *et al*, 1998; Joslyn and West, 2000; Newman *et al*, 2002). Recent studies, analysing molecular subtypes, suggest there is a higher frequency of the poor-prognosis basal subgroup (frequently referred to as triple negative) in young black American women (Carey *et al*, 2006).

Critics of the A-A data dispute the contributory impact of factors such as socioeconomic differences (Eley *et al*, 1994) and disparities in access to, and receipt of, health care between the ethnic groups (Dignam *et al*, 1997; Bickell *et al*, 2006). Although any such variations are more likely to contribute to outcome rather than age at presentation, the differences in health care systems between the USA and the UK have made it possible for us to control for these factors in a way that has not been possible in previously published studies.

Until now there have been no data on the patterns of breast cancer in British black women. Similarities with the A-A population have been assumed by most UK doctors, but this may well be an unwarranted assumption. It would seem prudent to review the biology of the disease in this group because of the potentially different genetic backgrounds between A-A and British black women. For example, use of population-specific alleles has shown European admixture to be considerably higher in African-Americans than in their Jamaican counterparts (Parra *et al*, 1998), and Afro-Caribbean people make up a substantial proportion of the British black population. Whether or not the British black population have a higher frequency of basal tumours, or if they have distinct molecular characteristics, certainly has never been addressed, although this could have major implications for breast cancer care in the UK, including screening and treatment protocols. Because ethnicity often has not been noted in many Registry databases, we have conducted a pilot study on a single hospital where up to 25% of the local referral population is black. Supposing our results can be extrapolated to the general black population in the UK, our findings could have implications for both the detection and treatment of breast cancer in this group of women. In more general terms, the differences we have detected between breast cancer in this group of British black women and the disease as reported in A-A women have significance for our understanding of its underlying biology.

## MATERIALS AND METHODS

Women presenting at the Homerton University Hospital in Hackney, East London, between 1994 and 2005, with a diagnosis of invasive breast cancer, were entered into a database following local ethics approval. Where possible, details of age at presentation, self-reported ethnicity, grade, lymph node status, stage, ER, PgR and ERBB2 (v-erb-b2 erythroblastic leukaemia viral oncogene homologue 2) status were recorded. From 2001 onward the hospital routinely recorded all incident breast cancers in the BASO database (British Association of Surgical Oncologists) and this is used currently in clinical practice. No such routine centralized data collection existed before 2001 and therefore the hospital computerized discharge summaries between 1994 and 2000 were searched. Histological diagnosis was confirmed by review of histology reports. Where ethnicity was undisclosed or histological diagnosis unconfirmed, patients were excluded. Only invasive breast cancer was included. The tumour specimens for each patient were retrieved from pathology archives and stained for ER, PgR and ERBB2 by immunohistochemistry (IHC) where this information was otherwise missing. Socioeconomic status was measured using the Index of Multiple Deprivation (IMD), a proxy for socioeconomic status, as determined by area of residence (Jordan *et al*, 2004). The IMD uses six domains to assess a given ward: income, employment, health deprivation and disability, education skills and training, housing and geographical access to services. Hackney was reported to be the most deprived area of London by IMD 2004.

For comparative purposes the details of all black patients presenting to the same hospital with cancer of any type during 2000–2007 were also recorded.

### Statistical methods

Age distributions of the black patients and white patients were compared using Poisson regression, adjusting for the different age distributions of the entire local populations. This was accomplished using the 2001 census figures for Hackney, London, where the majority of the patients resided (http://www.statistics.gov.uk/census, 2001). These were available only in three broad age groups, 0–15, 16–59 and 60 or more. Histological and biological features of the tumours were compared using logistic regression, adjusting for age in the first instance, and then for age and IMD. Survival analysis was by proportional hazards regression, also adjusting for age and IMD (Cox, 1972). We also tested for heterogeneity of results by tumour size and age, usually dichotomising age at a cut-off of 60 years, as this was the approximate median age in the two groups combined.

## RESULTS

A total of 445 patients with a new diagnosis of breast cancer made between 1994 and 2005 were identified. Nine white women and one black woman were removed from the cohort because histological review revealed a diagnosis of only ductal carcinoma *in situ* (DCIS). Sixteen white women were excluded because their initial diagnosis proved to have been before 1994. There were 126 women from other ethnic groups (5 Greek, 35 Jewish, 9 Turkish, 2 Chinese/Vietnamese, 3 Arabic and 19 Indian women), or where ethnicity was undisclosed, who were excluded from the cohort analysis. Data were obtained from 102 black women and 191 white British women.

**Figure 1:**
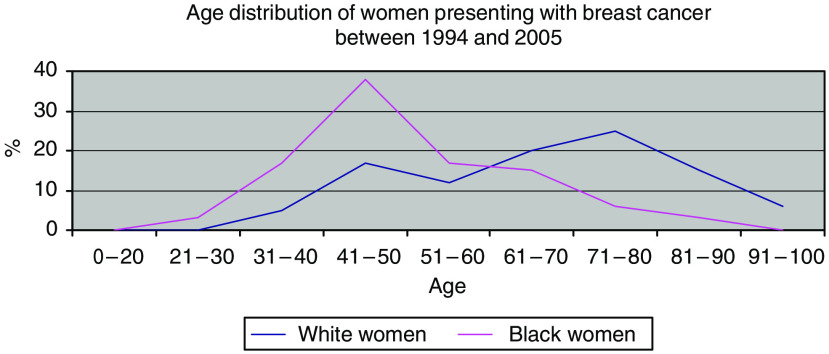
Age distribution of women presenting with breast cancer between 1994 and 2005.

The distributions of age at diagnosis in the black cohort and white cohort are demonstrated in Figure 1. This shows that the black patients were significantly younger (*P*=0.001), with a median age of 46 compared with 67 for the white patients. To address whether this difference in the age at presentation simply reflected differences in the age structure of the two ethnic populations locally, the patient cohorts were compared with the population census data of Hackney (Table 1). No significant difference between the black and the white population structures was identified, confirming that there is a true increase in the frequency of breast cancer in young black women. Moreover, no other common cancer in the same population of black women revealed a comparable increase in frequency in younger women (data not shown).

The pathological and biological features of the tumours in the two patient cohorts were compared (Table 2). Total numbers vary due to differing numbers of cases with missing data for each variable; however, black patients had a greater frequency of grade 3 tumours, lymph node-positive disease and negative ER and PgR status, compared with white women. They also had higher proportions of tumours of basal or triple negative status (as defined here by ER-negative, PgR-negative and ERBB2-negative status). The difference reached statistical significance only for histological grade (*P*=0.02). Results were unchanged when further adjusted for IMD.

There was borderline significant heterogeneity by age of the association of ethnicity with ER status (*P*=0.05) such that in patients aged under 60, the black patients were significantly more likely to have ER-negative disease (OR=2.36, 95% CI 1.06–5.00, *P*=0.03), but there was no significant difference in ER status by ethnicity in patients aged 60 years or above (OR=0.71, 95% CI 0.23–2.18, *P*=0.5). A similar heterogeneity of borderline significance was noted for basal status (*P*=0.09). In women aged under 60 years, black patients were more likely to have triple negative disease (OR=2.33, 95% CI 0.88–6.18), whereas in women aged 60 or more, there were no significant or suggestive differences between the two ethnic groups (OR=0.67, 95% CI 0.13–3.39). The ER and triple negative status by age group and ethnicity is shown in Table 3.

**Figure 2:**
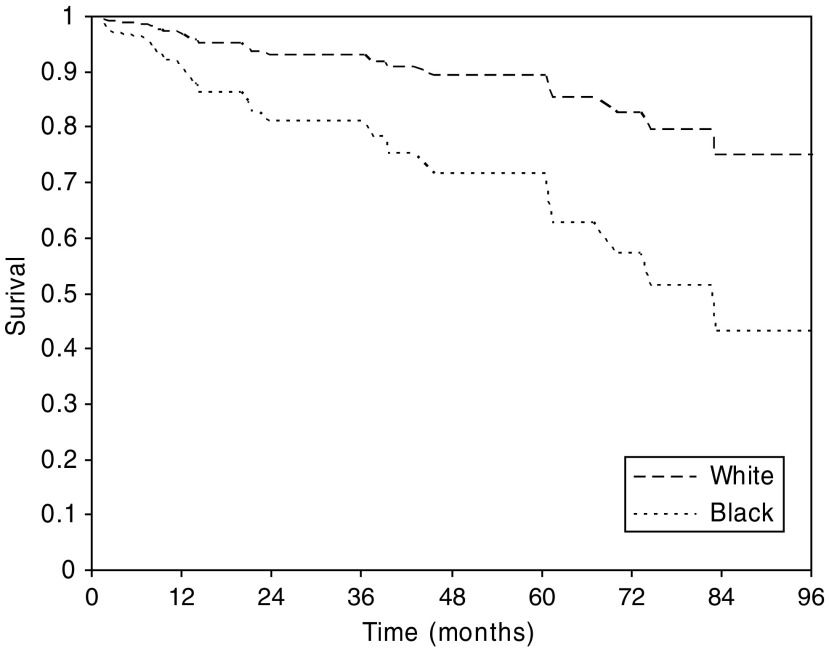
Age and IMD-adjusted estimated survival by ethnic group, for tumours of size 2 cm or less.

For overall survival analysis, there was an average follow-up of 3 years and a maximum of 12 years. The number of patient deaths by age group and ethnicity is shown in Table 4. Adjusting for age and IMD, no significant difference in survival was detected between black patients and white patients (HR=0.98, 95% CI 0.61–1.55, *P*=0.9). There was, however, significant heterogeneity of the effect of ethnic group in tumours of different sizes (*P*=0.002). In tumours of size 2 cm or less, black patients had poorer survival than white patients (HR=2.90, 95% CI 0.98–8.60, *P*=0.05), although for tumours greater than 2 cm, there was no significant or substantial difference in survival (HR=0.86, 95% CI 0.44–1.65, *P*=0.6) (Figure 2). The result was not changed substantially when further adjusted for grade and ER status. Survival by ethnicity for average age (61 years) and IMD score (46), in tumours of size 2 cm or less from the Cox regression, is shown in Figure 2.

## DISCUSSION

This study has demonstrated a substantial difference in age at presentation for breast cancer between white women and black women living in a geographically restricted deprived area of London. Black women presented on average 21 years younger than their white counterparts (a median age of 46 years), and this is earlier than the current threshold age of 50 years for entry to the UK National Health Service Breast Screening Programme (NHS BSP).

By selecting a single district hospital, the patients in this pilot study come from a limited geographical referral area, which controls, to a large extent, for the socioeconomic differences associated with risk of disease, accessibility to health resources and inequalities in receipt of treatment which might have accounted for these differences. This was further controlled for by generating an IMD score for each patient and adjusting for this in the analysis. This approach has restricted the number of cases that can be included in the study, but it has meant that the results are not a consequence of these variables.

Information on age of incidence of invasive breast cancer cases in London between 1994 and 2004 (41 792 women inclusive of all ethnic origins) is available. Twenty-five percent of all breast cancer cases in London presented at 45 years or younger compared with 45% of black women with breast cancer in our local population. It is crucial, therefore, to target this group of women to raise their awareness regarding the risks of breast cancer, the likelihood of early age at presentation and the importance of self-examination and early presentation with clinical signs.

The UK NHS BSP is offered to all women between 50 and 70 years, with an invitation to mammography on a 3 yearly basis. Alterations to the screening services offered to black populations might be considered to better reflect the incidence patterns for this group, much as it has been for those individuals with a family history deemed to be at risk of breast cancer at a younger age. Additional resources required for the screening of black women from age 40 or 45 would be modest, but there could be organizational difficulties in identifying the relevant population for invitation.

There has been much speculation regarding biological factors, which may underpin ethnic differences in breast cancer biology, presentation and outcome (Elledge *et al*, 1994; Joslyn and West, 2000). In our cohort, 62% of black women had grade 3 tumours compared with 42% of white women. Women under 50 years tend to have an increased rate of higher grade tumours. However, even after adjusting for age, there were significantly more grade 3 tumours in black women. Thus, it is not simply a consequence of the higher representation of younger women in the black cohort. Also, among those women with smaller tumours (⩽2 cm), black women were more than twice as likely to die of their disease (*P*=0.05). Review of the database has shown that black women received more adjuvant therapy (chemotherapy, radiotherapy and, where appropriate, hormone therapy) than their white counterparts. Thus, there is no evidence that observed differences are due to late presentation or inequalities in the receipt of therapy and, therefore, breast cancers arising in young black women appear to be biologically different; an effect not attributable simply to the young age of affected individuals.

It also is worth noting a particularly novel finding of our study that is, not only do British black women develop breast cancer at a younger age, but also smaller tumours (⩽2 cm) in young women show very different behaviour between the two ethnic groups. Tumours in younger women are considerably more aggressive in black women. Breast tumours in women over 60 years show similar behaviour regardless of ethnic origin. That the lymph node status and stage at presentation were similar between both ethnic groups also differs from reported findings in the A-A populations (Elledge *et al*, 1994; Newman *et al*, 2002).

Factors such as obesity, family history, low parity, later age at first full-term pregnancy, not breast feeding and long duration of reproductive period all are known to increase the risk of developing breast cancer (Collaborative Group on Hormonal Factors in Breast Cancer, 2002) and may differ significantly between ethnic groups. These risk factors have been associated with breast cancer in African women (Okobia *et al*, 2006) and may contribute to our observed differences to some extent.

Much of the work done in this area has attempted to try to explain the variation in outcome between A-A women and age- and stage-matched white Caucasians. Differences in receipt of optimal treatment have been cited as a possible cause (Dignam *et al*, 1997; Hershman *et al*, 2005). However, a pooled analysis of women treated within clinical trials, thus controlling for disease stage and receipt of treatment, still found significant differences in outcome for both pre- and post-menopausal A-A women (Albain *et al*, 2003). Such data suggest that biological features of the tumours are, at least partly, responsible for these ethnic differences (Selaru *et al*, 2004; Newman *et al*, 2006). Disease pattern variation between the ethnic groups is not explained by overexpression of the oncogene cerbB2+, as this does not differ significantly across racial groups (Carey *et al*, 2006).

Recent gene expression analyses of breast cancers have confirmed the existence of distinct molecular subgroups (Perou *et al*, 2000; Sorlie *et al*, 2003; Carey *et al*, 2006). There has been particular interest in the subgroup of basal-like tumours, which express many myoepithelium-associated genes, such as cytokeratin 5, and which are both cerbB2- and ER-negative and are associated with a poor prognosis (Turner *et al*, 2005). The basal phenotype is more common in breast cancers arising in young women and in BRCA-1-mutated cancers, to which patterns of disease in black women bear many similarities (Newman *et al*, 2002; Sorlie *et al*, 2003). Of note, A-A women do not appear to have a greater prevalence of high-risk BRCA mutations than the white Caucasian population (Frank *et al*, 2002; Olopade *et al*, 2003; Nanda *et al*, 2005), but the basal-like subtype is known to be more prevalent among young (premenopausal) A-A women with breast cancer (Carey *et al*, 2006). We noted it to be more prevalent, based on immunohistochemical analysis, in the British black population (22%), albeit at a much lower level than in the A-A population (39%). However, it also is apparent that the basal subtype is a heterogeneous group (Vincent-Salomon *et al*, 2007), and further analysis is required to more accurately define the precise nature of the tumours in these women. A detailed analysis of these tumours, using a more extensive panel of IHC markers to delineate the basal phenotype, is warranted. Of note, basal and ERBB2 subgroups have been found to respond better to 5-fluorouarcil, doxorubicin and cyclophosphamide chemotherapy relative to other breast cancer subgroups (Rouzier *et al*, 2005). Studies have shown that most triple negative tumours are included within this basal subset (Perou *et al*, 2000; Sorlie *et al*, 2001, 2003; van't Veer *et al*, 2002). These triple negative breast cancers are resistant to existing targeted treatments, such as hormonal treatments and trastuzumab, underscoring the clinical importance of defining new potential targets for treatment in this group. Interestingly, due to the characteristic defects in DNA repair seen in BRCA1 mutation-associated tumours, sensitivities to standard cytotoxic agents differ compared with other breast cancers (Bhattacharyya *et al*, 2000; Quinn *et al*, 2003). Novel targeted agents, being investigated in BRCA1/triple negative tumours, may also be of benefit in black women.

When specific gene expression signatures are anticipated to revolutionise the diagnosis and treatment of cancer, it may be important to recognise the distinct biological characteristics occurring within specific ethnic populations, and also the subtle differences between apparently similar populations, such as the A-A and British black populations, which might impact on these profiles.

In summary, we have shown that British black women, as often assumed by many UK clinicians, do share certain characteristics with their A-A counterparts with regard to breast cancer. There are although certain differences between these two groups, as exemplified by the aggressive behaviour of small tumours, which indicate that the two cohorts cannot be considered identical.

## Figures and Tables

**Table 1:** Age distribution of 293 breast cancer patients, and of the Hackney population, by ethnic group

	**Black**		**White**	
**Age group**	**Patients (%)**	**Population (%)**	**Patients (%)**	**Population (%)**
16–59	75 (74)	16 600 (86)	64 (34)	41 600 (80)
60+	27 (26)	2600 (14)	127 (66)	10 300 (20)

**Table 2:** Pathological and biological tumour features in black and white breast cancer patients

**Factor**	**Category**	**Black patient no. (%)**	**White patient no. (%)**	**Significance^a^**
Tumour size	⩽2 cm	39 (41)	64 (45)	0.2
	>2 cm	55 (59)	77 (55)	
	Total	94 (100)	141	
Node status	Negative	34 (35)	58 (41)	0.2
	Positive	62 (65)	85 (59)	
	Total	96 (100)	143 (100)	
Histological grade	1	6 (6)	18 (12)	0.02
	2	30 (32)	66 (46)	
	3	57 (62)	60 (42)	
	Total	93 (100)	144 (100)	
Oestrogen receptor status	Negative	32 (34)	34 (25)	0.2
	Positive	61 (66)	102 (75)	
	Total	93 (100)	136 (100)	
Progesterone receptor status	Negative	32 (36)	34 (25)	0.5
	Positive	58 (64)	102 (75)	
	Total	90 (100)	136 (100)	
Her2/neu status	Negative	56 (66)	83 (65)	0.6
	Positive	29 (34)	44 (35)	
	Total	85 (100)	127 (100)	
Triple negative status	Negative	63 (78)	99 (85)	0.2
	Positive	18 (22)	17 (15)	
	Total	81 (100)	116 (100)	

a Adjusted for age.

**Table 3:** ER status and triple negative status by age and ethnicity

**Factor**	**Age group**	**Category**	**Black patient no. (%)**	**White patient no. (%)**	**Significance**
ER status	<60	Negative	27 (39)	13 (21)	0.03
		Positive	43 (61)	48 (79)	
		Total	70 (100)	61 (100)	
	60+	Negative	5 (22)	21 (28)	0.5
		Positive	18 (78)	54 (72)	
		Total	23 (100)	75 (100)	
Triple negative status	<60	Negative	48 (75)	49 (88)	0.09
		Positive	16 (25)	7 (12)	
		Total	64 (100)	56 (100)	
	60+	Negative	15 (88)	50 (83)	0.6
		Positive	2 (12)	10 (17)	
		Total	17 (100)	60 (100)	

ER=oestrogen receptor.

**Table 4:** Patients and deaths by age and ethnicity

**Ethnicity**	**Age group**	**Patients**	**Deaths (% of patients)**
Black patients	<40	19	5 (26)
	40–49	38	8 (21)
	50–59	18	10 (56)
	60–69	14	6 (42)
	70+	13	5 (38)
White patients	<40	8	2 (25)
	40–49	32	5 (16)
	50–59	24	7 (29)
	60–69	39	17 (44)
	70+	88	61 (69)
